# Influenza outbreak control practices and the effectiveness of interventions in long-term care facilities: a systematic review

**DOI:** 10.1111/irv.12203

**Published:** 2013-11-07

**Authors:** Kaitlin Rainwater-Lovett, Kevin Chun, Justin Lessler

**Affiliations:** Department of Epidemiology, Johns Hopkins Bloomberg School of Public HealthBaltimore, MD, USA

**Keywords:** Attack rate, influenza, intervention, long-term care facility

## Abstract

**Background:**

Evaluation of influenza control measures frequently focuses on the efficacy of chemoprophylaxis and vaccination, while the effectiveness of non-pharmaceutical interventions (NPI) receives less emphasis. While influenza control measures are frequently reported for individual outbreaks, there have been few efforts to characterize the real-world effectiveness of these interventions across outbreaks.

**Objectives:**

To characterize influenza case and outbreak definitions and control measures reported by long-term care facilities (LTCFs) of elderly adults and estimate the reduction in influenza-like illness (ILI) attack rates due to chemoprophylaxis and NPI.

**Methods:**

We conducted a literature search in PubMed including English-language studies reporting influenza outbreaks among elderly individuals in LTCFs. A Bayesian hierarchical logistic regression model estimated the effects of control measures on ILI attack rates.

**Results:**

Of 654 articles identified in the literature review, 37 articles describing 60 influenza outbreaks met the inclusion criteria. Individuals in facilities where chemoprophylaxis was used were significantly less likely to develop influenza A or B than those in facilities with no interventions [odds ratio (OR) 0·48, 95% CI: 0·28, 0·84]. Considered by drug class, adamantanes significantly reduced infection risk (OR 0·22, 95% CI: 0·12, 0·42), while neuraminidase inhibitors did not show a significant effect. Although NPI showed no significant effect, the results suggest that personal protective equipment may produce modest protective effects.

**Conclusions:**

Our results indicate pharmaceutical control measures have the clearest reported protective effect in LTCFs. Non-pharmaceutical approaches may be useful; however, most data were from observational studies and standardized reporting or well-conducted clinical trials of NPI are needed to more precisely measure these effects.

## Background

It is estimated that 90% of deaths due to influenza infection occur in individuals aged 65 or older.^1^ Many older individuals live in long-term care facilities (LTCFs), where they have frequent interactions with other residents and nursing staff, but few interactions with the outside world. Outbreaks of influenza in LTCFs are of particular concern as residents are at high risk of severe complications and less likely to be protected by vaccination.^2^ In 1999, the Society for Healthcare Epidemiology of America (SHEA) published six recommendations for the prevention and control of influenza outbreaks in LTCFs: (i) mass vaccination of unvaccinated residents and staff; (ii) use of prophylactic treatment with adamantanes (amantadine or rimantadine); (iii) decreasing contact between residents; (iv) re-emphasis on compliance with handwashing; (v) furlough of sick staff; and (vi) cohorting of residents.^3^ Current guidelines by the US Centers for Disease Control (CDC)^4^ draw heavily on these measures with updated recommendations for use of neuraminidase inhibitors (oseltamivir or zanamivir) rather than adamantanes.

Most work evaluating influenza control measures has focused on vaccination and antivirals, reported direct vaccine effectiveness in a single LTCF after a seasonal outbreak,^5^–^16^ or used a randomized controlled trial (RCT) design where individuals or facilities randomized to receive an intervention were compared with those receiving a placebo or the standard of care.^17^–^21^ While these studies are essential to establishing the effectiveness of interventions, several gaps in our understanding of the effectiveness of control measures remain. First, when considering a single randomized population, we only measure the direct effect of an intervention, potentially underestimating its true population effectiveness. Second, RCTs provide an efficacy estimate in a controlled setting, but do not necessarily reflect the effectiveness of a policy in real-world situations where resources, implementation and patient populations vary. Finally, inadequate attention has been paid to non-pharmaceutical interventions (NPI) such as personal protective equipment (PPE) and social distancing that have widespread use, are often used in conjunction with other approaches and prevent transmission of antiviral-resistant influenza strains and other co-circulating respiratory pathogens.

Because LTCFs provide a set of similar populations that are relatively closed to the outside world, they provide an opportunity to evaluate the effectiveness of interventions that are difficult to characterize across more heterogeneous populations. Here, we report the results of a systematic review and meta-analysis of the effectiveness of influenza control measures implemented in LTCFs. To more effectively capture how LTCFs respond to influenza-like illness (ILI), we characterize case and outbreak definitions and the range of outbreak responses that have been reported in the literature. We estimate the effectiveness of pharmaceutical and NPI using robust statistical techniques.

## Methods

### Search strategy and selection criteria

We conducted a systematic review in accordance with the PRISMA statement.^22^ A literature search was conducted during September 2011 in the PubMed database using the following search phrase: (influenza) AND (‘long-term care’ OR ‘long-term care’ OR ‘assisted living’ OR ‘nursing home’ OR ‘nursing homes’). LTCFs were defined as any residential environment that housed older adults or elderly individuals with the assistance of medical staff, and included facilities referred to as ‘assisted living’ or ‘nursing homes’ but excluded community centers and daytime-only facilities serving older adults living in the outside community. Two individuals (KRL and KC) screened all abstracts independently using Microsoft Access databases and discrepancies were resolved by discussion and consensus.

Studies of any design reporting influenza outbreaks among elderly individuals in LTCFs were considered for inclusion. During full text review, articles were included if they contained the number of influenza cases occurring on a specific date or within a 3-day period, such as through an epidemic curve or a line list of symptom onset dates. Articles were excluded if they did not contain information on influenza infections of humans, elderly individuals and individuals in LTCFs. Articles written in a language other than English or without an abstract were excluded.

### Data abstraction

Data were abstracted using standardized forms in Microsoft Excel and included the population type (residents, staff or both), age range, influenza case definition, influenza outbreak definition, number of influenza cases, number of residents and beds in the LTCF, staff size, geographic location, start and end dates of the outbreak, start and end dates of the interventions, proportions of residents and staff receiving seasonal influenza vaccine, use of chemoprophylaxis (amantadine, rimantadine, oseltamivir, and zanamivir), use of reactive vaccination, type and policy of NPI use (droplet precautions, hand hygiene, isolation or cohorting, masks), and policies regarding ill staff and visitor restrictions.

### Statistical analysis

Attack rates were calculated by dividing the number of reported cases by the number of individuals at-risk for influenza viral infection. The population considered ‘at-risk’ was determined based on the group of individuals who were under observation in the report (e.g., all residents, all residents and staff, or residents who agreed to be monitored in an observational study). Number of beds was substituted for the number of individuals ‘at-risk’ when no other estimate of population size was provided. Student's t-test and analysis of variance were used to compare mean attack rates between intervention types, and exact 95% confidence intervals were calculated using the Poisson distribution. The effects of vaccination rates over time and on attack rates were evaluated using linear regression.

We fit a hierarchical binomial model to the number of cases infected given the estimated population at-risk in each outbreak using a Bayesian framework with non-informative priors. Facility-specific attack rates were modeled as a logistic regression, where each facility had a unique intercept term drawn from a normal distribution with estimated mean and variance. Odds ratios (OR) estimate the difference in the odds of becoming an influenza case in a facility with a particular policy versus a facility where no intervention was implemented. This approach allows us to use information contributed by each individual in the outbreak, while appropriately accounting for variations in baseline risk across institutions. Statistical analysis was conducted in R version 2.13 and OpenBUGS.^23^,^24^

## Results

**Figure 1:**
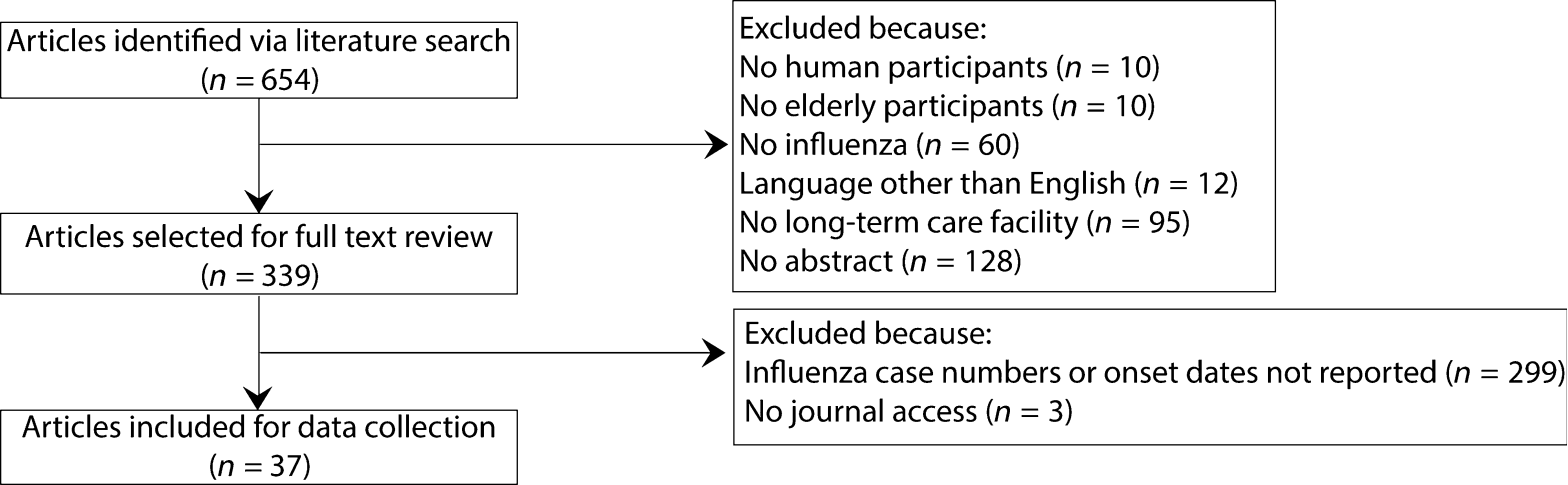
Article selection process. *n* = number of articles.

A total of 654 articles were identified in the literature search. Three-hundred and fourteen articles were excluded after reviewing abstracts, and 303 articles were excluded after full text review (Figure 1). Thirty-seven articles described 60 outbreaks occurring between 1980 and 2011 (Table 1), and 8 articles (21%) reported more than one outbreak. The reported outbreaks occurred in Australia (*n* = 4), Belgium (*n* = 1), Canada (*n* = 7), England (*n* = 3), France (*n* = 3), Japan (*n* = 4), Singapore (*n* = 1), Taiwan (*n* = 1), and the United States (*n* = 36). Fifty-one outbreaks (85%) consisted of only influenza A cases, seven (12%) consisted of only influenza B cases, and two (3%) consisted of cases of influenza A and B. The median number of cases in each outbreak was 28 (range: 7, 139) and a median of 128 individuals was at-risk of influenza (range: 28, 729).

**Table 1:** Chemoprophylaxis and non-pharmaceutical interventions for influenza outbreak control in long-term care facilities by article

Author (year)*	Number of outbreaks included in review	Numbers of cases/individuals at-risk	Chemo prophylaxis	Personal protective equipment	Social distancing
Arden *et al*. (1988)^29^	1	14/55	A	–	–
Arroyo *et al*. (1984)^5^	1	89/176	–	H, M	N, V
Bowles *et al*. (2002)^31^	4	95/413	A, O	–	–
Burette *et al*. (2009)^42^	1	32/62	–	–	–
Bush *et al*. (2004)^32^	1	26/91	O	D	I, N, T, V
Chang *et al*. (2008)^6^	1	23/55	O	–	–
Coles *et al*. (1992)^7^	1	55/270	–	–	–
Degelau *et al*. (1992)^56^	1	22/140	A	–	–
Dindinaud *et al*. (1993)^43^	1	65/116	–	–	–
Drinka *et al*. (1999)^44^	5	223/3560	A, R	–	–
Drinka *et al*. (2000)^45^	4	152/718	R, Z	–	–
Drinka and Haupt (2007)^46^	1	15/50	O, R	D	I
Ferson *et al*. (2004)^37^	1	35/69	–	–	I, N, V
Gaillat *et al*. (2008)^30^	1	38/129	O	M	I
Goodman *et al*. (1982)^47^	1	30/120	–	–	I, V
Hall *et al*. (1981)^48^	1	129/359	–	H	I
Horman *et al*. (1986)^49^	1	76/170	–	–	–
Infuso *et al*. (1996)^8^	1	43/66	–	–	–
Lee *et al*. (2000)^9^	1	69/176	A, Z	D	N, V
Libow *et al*. (1996)^50^	1	139/499	A	–	I
Mast *et al*. (1991)^51^	2	139/1162	A	–	N, V
Mathur *et al*. (1980)^38^	1	25/354	–	H, M	I, V
Meiklejohn *et al*. (1989)^10^	1	40/98	–	–	–
MMWR (1993)^52^	1	99/224	–	–	–
Morens and Rash (1995)^53^	1	11/39	A	D	I, V
Murayama *et al*. (1999)^11^	2	66/256	–	–	–
Oguma *et al*. (2011)^54^	2	24/312	–	H, M	I
Parker *et al*. (2001)^12^	1	28/286	O	–	I
Peters *et al*. (1989)^27^	1	15/140	A	–	–
Read *et al*. (2000)^55^	3	85/211	–	–	N, V
Schilling *et al*. (2004)^28^	1	68/721	A	H, M	I, V
Schilling *et al*. (1998)^18^	9	66/250	R, Z*	–	–
Seale *et al*. (2009)^13^	1	22/89	O	–	I
Staynor *et al*. (1994)^14^	1	22/88	A	–	I, N, V
Strassburg *et al*. (1986)^15^	1	46/87	–	–	–
Taylor *et al*. (1992)^16^	1	59/137	–	–	–
Win *et al*. (2010)^41^	1	19/180	–	–	–

A, amantadine; O, oseltamivir; R, rimantadine; Z, zanamivir. D, droplet precautions; H, hand hygiene; I, isolation or cohorting; M, masks; N, no new admissions; T, ward transfer restrictions; V, visitor restriction.

* Chemoprophylaxis was not used in one outbreak reported in this article.

### Influenza case definitions

Fifty-seven (95%) of 60 outbreaks reported a case definition for identifying influenza in the facility (Table S1). The Centers for Disease Control and Prevention define ILI as a temperature of at least 100°F (37·7°C) and cough or sore throat in the absence of a known cause other than influenza.^25^ Seven (12%) of 57 outbreaks with case definitions used a variation of this definition by also including individuals with coryza, sneezing or rhinorrhea, while 18 outbreaks (32%) had less specific definitions requiring a fever and at least one additional sign or symptom such as malaise or pneumonia. Twenty-one outbreaks (37%) did not require fever but included fever among possible signs defining an influenza case. While all studies used laboratory testing to establish influenza as the cause of the outbreak, eleven outbreaks (19%) required diagnostic confirmation of each case through the use of rapid diagnostic testing, fourfold seroconversion, viral culture or polymerase chain reaction (PCR). Three outbreaks required both laboratory confirmation and a consistent clinical presentation to identify cases.

### Epidemic thresholds

Fourteen outbreaks (23%) reported a facility policy defining an influenza outbreak (Table S1). Three outbreaks defined an influenza outbreak as the detection of two or more cases of ILI within 72 hours in a single residential unit. Two of these outbreaks required at least one positive rapid influenza diagnostic test among the ILI cases. The remaining 11 outbreaks declared an outbreak when an attack rate of at least 10% was observed within a 7-day period, with nine outbreaks requiring influenza viral isolation during the previous 7 days.

### Antiviral prophylaxis

Chemoprophylaxis was defined as offering antiviral drugs to asymptomatic individuals in the facility. Forty (67%) of the 60 outbreaks used prophylactic antiviral drugs (Table 1), 19 of which used at least two drugs. Of the 40 outbreaks, 34 (85%) consisted of only influenza A cases, two (5%) included cases of influenza A and influenza B, and 4 (10%) consisted of only influenza B cases.

Amantadine was used in 19 (56%) of the 34 influenza A only outbreaks and one (50%) of the two influenza A/B outbreaks. Amantadine was the only antiviral drug used in nine outbreaks and was used alongside rimantadine (*n* = 4), oseltamivir (*n* = 5) and zanamivir (*n* = 1) (Table S2). Rimantadine was used in 14 (40%) of the influenza A only outbreaks and one (50%) of the two influenza A/B outbreaks. Rimantadine was used alone in two outbreaks and was used with zanamivir in eight outbreaks (Table S2). None of the influenza B only outbreaks used amantadine or rimantadine, as is consistent with lack of pharmacologic activity by adamantanes for influenza B.^26^

Compliance and side effects were typically reported by article. Compliance with amantadine use was reported by one article,^27^ which described staff prophylaxis as less than half of staff members taking ≥70% of the prophylactic regimen. Resident compliance was not reported by any articles. Five articles (13%) reported discontinuation of amantadine by 11/111 (10%) residents reporting side effects.^7^,^14^,^25^–^29^ Side effects were wide ranging with agitation, anorexia, depression, fatigue, and gastrointestinal symptoms most frequently reported.

Twenty-five (63%) of the 40 outbreaks offered neuraminidase inhibitors (Table S2): 14 used oseltamivir (11 influenza A outbreaks, 1 A/B, 2 B only) and 11 used zanamivir (nine influenza A, two B only). Only two outbreaks used zanamivir as the sole antiviral drug. One influenza A outbreak used amantadine, rimantadine, and zanamivir. Oseltamivir and zanamivir were never used in the same outbreak.

Refusal of oseltamivir prophylaxis was reported for 3/89 (3%) and 2/129 (2%) residents in two articles,^13^,^30^ and two articles each reported refusal by one patient after 2 and 3 days of prophylaxis without explanation.^12^,^31^ Side effects of oseltamivir resulting in discontinuation included difficulty swallowing (*n* = 1), nausea (*n* = 2), and vomiting (*n* = 2).^12^,^13^,^31^,^32^ Zanamivir was refused by 11/130 (8%) residents in one study and inhalations were difficult for 29/130 (22%) residents.^9^

### Vaccination

**Figure 2:**
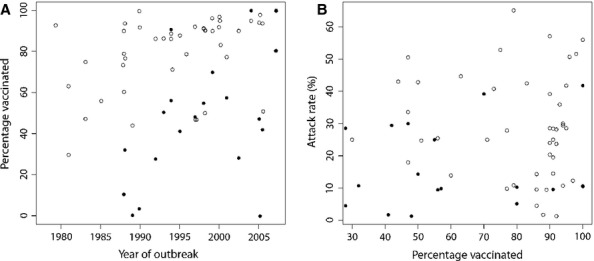
(A) Percentage of vaccinated long-term care facility residents (open circles) and staff (dark circles) over time in 47 and 21 influenza outbreaks reporting vaccination rates, respectively. (B) Percentage of vaccinated residents (open circles) and staff (dark circles) and influenza-like illness attack rates.

Prophylactic vaccination was defined as offering seasonal trivalent inactivated or live intranasal influenza vaccine to residents and/or staff prior to the identification of influenza cases in the facility. Institutions had prophylactically vaccinated a median of 89% of residents (IQR: 72%, 92%) and 48% of staff (IQR: 28%, 70%) during the most recent influenza season among 47 and 21 outbreaks, respectively. Thirty-four outbreaks (72%) reported ≥75% of residents vaccinated against seasonal influenza, while only five outbreaks (21%) reported this proportion of vaccinated staff. Over time, vaccination rates of staff and residents increased by 2·3% and 0·84% per year (*P* = 0·016 and *P* = 0·018) (Figure 2a), but increased staff and resident vaccination rates were not associated with decreased attack rates (*P* = 0·46 and *P* = 0·07) (Figure 2b).

Reactive vaccination was defined as offering influenza vaccine to asymptomatic individuals after the identification of influenza cases in the facility. Only four (7%) of the 60 outbreaks reported reactive vaccination as an outbreak control measure: in two outbreaks, vaccination was offered or recommended to staff and residents; in one outbreak, all unimmunized residents were vaccinated; and in one outbreak, vaccination was offered to staff. The number or percentage of individuals receiving reactive vaccination was not reported in any outbreak.

### Non-pharmaceutical interventions (NPI)

Physical measures attempting to reduce influenza transmission that did not require drugs or vaccines such as PPE and social distancing were defined as NPI (Table 1). Glove and mask use, hand hygiene, and droplet precautions were considered PPE and were reported by 19 (32%) of 60 outbreaks.

Social distancing was reported by 23 outbreaks (38%) and included patient isolation and restrictions on staff, visitors, admissions and ward transfers. Isolation was defined as restriction of movement within the facility by symptomatic individuals through the use of room, unit, or ward quarantine or cohorting and was reported by 13 outbreaks (22%). Two outbreaks (3%) reported the length of required isolation as a period of 5 days or for the duration of symptoms.

Visitor and staff restriction was reported by 14 of 60 outbreaks (23%). Four outbreaks (7%) reported policies for symptomatic staff during influenza outbreaks, which emphasized taking sick leave until symptom resolution or 5 days post-symptom onset (Table S3). Eleven outbreaks (18%) reported policies restricting visitors during influenza outbreaks (Table 1), including six outbreaks advising visitors of ongoing outbreaks or asking visitors to postpone visits (Table S3).

### Attack rates

**Figure 3:**
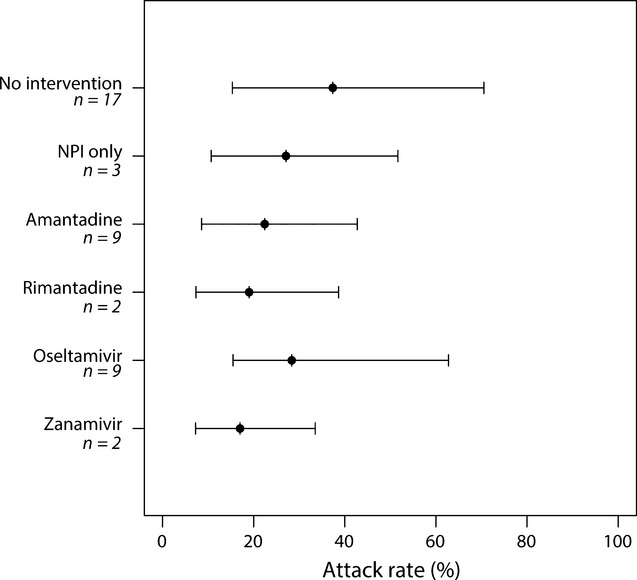
Unadjusted mean attack rates (95% CI) from influenza outbreaks in long-term care facilities by intervention method. Outbreaks do not sum to 60 as 19 outbreaks used ≥2 antiviral drugs for prophylaxis. Non-pharmaceutical interventions were used in conjunction with amantadine (*n* = 4 outbreaks), oseltamivir (*n* = 6 outbreaks) and zanamivir (*n* = 2 outbreaks). CI = confidence interval; *n* = number of outbreaks.

Influenza attack rates for the 60 outbreaks ranged from 1·3% to 65% with an unadjusted mean of 28%. Among 17 outbreaks that did not implement intervention measures, an unadjusted mean attack rate of 41% (95% CI: 24, 51) was observed, which did not differ from three outbreaks using only PPE [30% (95% CI: 19, 37)] or from 21 outbreaks using a single antiviral drug [25% (95% CI: 14%, 29%)] (Figure 3). Among 21 outbreaks using a single antiviral drug, unadjusted mean attack rates did not differ significantly between drugs (*P* = 0·44) and ranged from 17% (95% CI: 7, 34) for zanamivir to 29% (95% CI: 25, 42) for oseltamivir (Figure 3).

After adjusting for NPI, use of any antiviral drug halved attack rates among outbreaks consisting of influenza A cases [OR: 0·52 (95% CI: 0.29, 0·93)] and influenza A and B cases [OR: 0·48 (95% CI: 0·28, 0·84)] compared with outbreaks that did not implement influenza control measures (Table 2). More specifically, the use of adamantanes produced statistically significant protective effects among outbreaks with cases of influenza A [OR: 0·33 (95% CI: 0·17, 0·62)] and influenza A and B [OR: 0·27 (95% CI: 0·14, 0·48)]. Combined use of adamantanes and neuraminidase inhibitors consistently demonstrated protective effects, although not statistically significant, but surprisingly neuraminidase inhibitors alone did not (Table 2). Social distancing and PPE were not associated with significant changes in influenza attack rates (Table 2).

**Table 2:** Odds ratios (95% CI) of the effect of influenza interventions on attack rates among outbreaks consisting of influenza A only (Models 1 and 2) and outbreaks of influenza A or B (Models 3 and 4) in long-term care facilities, considering antiviral interventions together (Models 1 and 3) and as independent interventions (Models 2 and 4)

	Influenza A outbreaks (*n* = 51)		Influenza A or B outbreaks (*n* = 60)	
		
	Model 1	Model 2	Model 3	Model 4
No intervention	Reference*	Reference	Reference	Reference
Personal protective equipment	0·75 (0·33, 1·61)	0·53 (0·25, 1·10)	0·99 (0·49, 1·93)	0·63 (0·33, 1·19)
Social distancing	1·05 (0·53, 2·16)	1·35 (0·72, 2·62)	1·07 (0·58, 1·90)	1·31 (0·78, 2·18)
Any antiviral drug	**0·52 (0.29, 0·93)**	–	**0·48 (0·28, 0·84)**	–
Adamantanes	–	**0·33 (0·17, 0·62)**	–	**0·27 (0·14, 0·48)**
Neuraminidase inhibitors	–	1·55 (0·62, 3·98)	–	1·27 (0·56, 2·76)
Adamantanes and Neuraminidase inhibitors	–	0·64 (0·35, 1·28)	–	0·57 (0·31, 1·03)
Influenza B	–	–	0·55 (0·27, 1·15)	**0·39 (0·20, 0·82)**

CI, confidence interval; *n*, number of outbreaks.

Point estimates and 95% CIs were derived from hierarchical binomial models and are interpreted as the difference in the odds of becoming an influenza case in a facility with a particular policy versus a facility where no intervention was implemented.

Bolded estimates indicate statistically significant effects (*P* < 0·05).

* Reference indicates that outbreaks using ‘No intervention’ served as the comparison group for calculating odds ratios.

## Discussion

Antiviral prophylaxis significantly reduced influenza attack rates, reducing the odds of developing influenza by 50% among LTCF residents. While our results were consistent with a protective effect of PPE, this effect was not statistically significant. In these 60 outbreaks, we noted an emphasis on the use of antiviral prophylaxis and prophylactic vaccination in comparison with other SHEA-recommended influenza control measures,^3^ perhaps reflecting biases in what is considered important to report in the literature. We also found that attack rates in outbreaks of influenza B were consistently lower than for influenza A.

The best case definition for ILI is the source of much debate in the literature.^31^–^33^,^36^ In the outbreaks reviewed, the current ILI definition put forth by the CDC appears to be serving as a rough guideline.^25^ While different case definitions can lead to different attack rates, we included ILI with laboratory-confirmed influenza cases as our primary outcome to reflect current practices in LTCFs. All outbreaks included in this review confirmed influenza virus as the causative organism of at least the first case of ILI in the facility, and additional cases with similar clinical presentation were then assumed to be caused by a related influenza virus. We acknowledge this may have resulted in the inclusion of cases caused by other co-circulating respiratory pathogens, as were identified in a few studies here^37^,^38^; however, incorporating only laboratory-confirmed influenza cases would likely exclude influenza-infected individuals as diagnostic tests typically demonstrate sensitivities of less than 100% and have been shown to frequently misclassify elderly individuals.^39^

Many articles described high levels of prophylactic vaccination and reported specific chemoprophylaxis for residents, which have demonstrated effectiveness in other studies,^17^,^20^,^21^ but we are unaware of studies describing or evaluating the effectiveness of NPI across multiple influenza outbreaks in LTCFs. Basic measures like hand hygiene and droplet precautions are essential for reducing respiratory virus transmission,^40^ particularly in the early stages of an outbreak. The length of time between reactive vaccination and development of protective immunity or receipt of antiviral drugs and decreased viral shedding may not be sufficient to produce as rapid a decline in the number of incident infections as NPI. A systematic review on the prevention of respiratory viral transmission demonstrated protective effects of NPI but did not specifically assess influenza virus transmission.^40^ Furthermore, vaccination and chemoprophylaxis do not protect against all influenza viruses due to vaccine strain mismatches and antiviral-resistant strains. We suspect the lack of statistically significant protective effects of PPE and social distancing was the result of broad definitions as NPI were rarely reported in detail.

The effects of reporting biases are important to consider as many observational studies were incorporated into this analysis. First, we noted an outcome reporting bias, whereby authors consistently reported results related to vaccination and chemoprophylaxis but may have overlooked reporting NPI such as basic hygiene and isolation measures, which would contribute to NPI effect estimates. Second, NPI effectiveness may be underestimated due to LTCFs with good infection control practices experiencing fewer outbreaks or lower case numbers that would not be presented in the literature. Finally, intervention studies with null results may be under-represented in the literature due to both reporting and publication biases.

Resistance by influenza strains to adamantanes resulted in changes to antiviral guidelines and their use is no longer recommended in the United States.^4^ Among the articles reviewed in the present study, neuraminidase inhibitors were frequently used in conjunction with adamantanes, reducing our statistical power to estimate their independent effect. This difference may simply reflect statistical power or secular trends in influenza attack rates in LTCFs (outbreaks utilizing neuraminidase inhibitors generally occurred more recently). Randomized controlled trials suggest neuraminidase inhibitors should be effective: prior to widespread circulation of adamantane-resistant influenza viruses, one randomized study in a LTCF demonstrated zanamavir was more effective than rimantadine^18^; and a 2006 systematic review found that amantadine and neuraminidase inhibitors had similar prophylactic efficacy against symptomatic influenza, while rimantidine did not show a clear protective effect.^17^ A recent cluster RCT demonstrated lower attack rates and shorter mean duration of influenza outbreaks in LTCFs using neuraminidase inhibitors for both treatment and prophylaxis compared to treatment alone,^21^ suggesting increased use reduced transmission.

The inability to adjust for confounding factors, such as the health status of residents under study, discrepancies in intervention implementation across outbreaks, or differences in living conditions, was a limitation of this analysis. The article selection criteria allowed for inclusion of outbreaks occurring in assisted living facilities and nursing homes, which typically house individuals of different functional abilities. Only two outbreaks occurred among residents living exclusively in an assisted living facility^41^,^42^, and one outbreak occurred in a combined assisted living-nursing home facility,^32^ which were consistent with the remaining 58 outbreaks that occurred among residents of nursing homes. Moreover, our results appeared robust as case definitions were not associated with attack rates, and prophylactic vaccination of residents and staff was not related to antiviral use. Antiviral compliance and side effects were not frequent or consistent across articles.

This analysis provides further evidence that antiviral prophylaxis is one of the most effective ways to control influenza in populations at high risk of complications and where vaccine efficacy is reduced. Although prophylactic vaccination rates of staff and residents increased over time, sensitivity analyses adjusting for the proportion of vaccinated residents in the hierarchical models did not affect inferences of other control measures, and we found no evidence that higher vaccination rates were associated with a reduction in attack rates, suggesting low vaccine effectiveness in LTCF residents. More focus on developing a body of evidence on the most effective use of NPI among older, high-risk populations is clearly needed. While antiviral prophylaxis is highly effective, it may fail in the face of a novel, resistant strain. In such a scenario, NPI will be our only option for control and the importance of understanding which measures are most effective, and how effective they are, is paramount.

## Supporting Information

**Table S1.** Influenza case and outbreak definitions. **Table S2.** Number of influenza A outbreaks using one or two forms of chemoprophylaxis. **Table S3.** Staff and visitor policies for influenza outbreak control.

## References

[1] Thompson WW, Shay DK, Weintraub E (2003). Mortality associated with influenza and respiratory syncytial virus in the United States. JAMA.

[2] Goodwin K, Viboud C, Simonsen L (2006). Antibody response to influenza vaccination in the elderly: a quantitative review. Vaccine.

[3] Bradley SF (1999). Prevention of influenza in long-term-care facilities. Long-Term-Care Committee of the Society for Healthcare Epidemiology of America. Infect Control Hosp Epidemiol.

[4] US Centers for Disease Control and Prevention Interim guidance for influenza outbreak management in long-term care facilities. http://www.cdc.gov/flu/professionals/infectioncontrol/ltc-facility-guidance.htm.

[5] Arroyo JC, Postic B, Brown A, Harrison K, Birgenheier R, Dowda H (1984). Influenza A/Philippines/2/82 outbreak in a nursing home: limitations of influenza vaccination in the aged. Am J Infect Control.

[6] Chang YM, Li WC, Huang CT (2008). Use of oseltamivir during an outbreak of influenza A in a long-term care facility in Taiwan. J Hosp Infect.

[7] Coles FB, Balzano GJ, Morse DL (1992). An outbreak of influenza A (H3N2) in a well immunized nursing home population. J Am Geriatr Soc.

[8] Infuso A, Baron S, Fauveau H, Melon M, Fleury H, Desenclos JC (1996). Value of influenza vaccine during an outbreak of influenza A in a nursing home, Pyrenees Atlantiques, France, November-December 1995. Euro Surveill.

[9] Lee C, Loeb M, Phillips A (2000). Zanamivir use during transmission of amantadine-resistant influenza A in a nursing home. Infect Control Hosp Epidemiol.

[10] Meiklejohn G, Hoffman R, Graves P (1989). Effectiveness of influenza vaccine when given during an outbreak of influenza A/H3N2 in a nursing home. J Am Geriatr Soc.

[11] Murayama N, Suzuki H, Arakawa M, Nerome K, Mizuta K, Kameyama K (1999). Two outbreaks of influenza A (H3N2) in a Japanese nursing home in the winter of 1996-1997, with differing vaccine efficacy. Tohoku J Exp Med.

[12] Parker R, Loewen N, Skowronski D (2001). Experience with oseltamivir in the control of a nursing home influenza B outbreak. Can Commun Dis Rep.

[13] Seale H, Weston KM, Dwyer DE (2009). The use of oseltamivir during an influenza B outbreak in a chronic care hospital. Influenza Other Resp Viruses.

[14] Staynor K, Foster G, McArthur M, McGeer A, Petric M, Simor AE (1994). Influenza A outbreak in a nursing home: the value of early diagnosis and the use of amantadine hydrochloride. Can J Infect Control.

[15] Strassburg MA, Greenland S, Sorvillo FJ, Lieb LE, Habel LA (1986). Influenza in the elderly: report of an outbreak and a review of vaccine effectiveness reports. Vaccine.

[16] Taylor JL, Dwyer DM, Coffman T, Groves C, Patel J, Israel E (1992). Nursing home outbreak of influenza A (H3N2): evaluation of vaccine efficacy and influenza case definitions. Infect Control Hosp Epidemiol.

[17] Jefferson T, Demicheli V, Rivetti D, Jones M, Di PC, Rivetti A (2006). Antivirals for influenza in healthy adults: systematic review. Lancet.

[18] Schilling M, Povinelli L, Krause P (1998). Efficacy of zanamivir for chemoprophylaxis of nursing home influenza outbreaks. Vaccine.

[19] Monto AS, Ohmit SE, Hornbuckle K, Pearce CL (1995). Safety and efficacy of long-term use of rimantadine for prophylaxis of type A influenza in nursing homes. Antimicrob Agents Chemother.

[20] Jefferson T, Rivetti D, Rivetti A, Rudin M, Di PC, Demicheli V (2005). Efficacy and effectiveness of influenza vaccines in elderly people: a systematic review. Lancet.

[21] Booy R, Lindley RI, Dwyer DE (2012). Treating and preventing influenza in aged care facilities: a cluster randomised controlled trial. PLoS ONE.

[22] Moher D, Liberati A, Tetzlaff J, Altman DG (2009). Preferred reporting items for systematic reviews and meta-analyses: the PRISMA statement. PLoS Med.

[23] R Core Team R: A language and environment for statistical computing. http://www.R-project.org.

[24] Thomas A, O'Hara B, Ligges O, Sturtz S (2006). Making BUGS Open. R News.

[25] US Centers for Disease Control and Prevention Overview of influenza surveillance in the United States. http://www.cdc.gov/flu/weekly/overview.htm.

[26] Davies WL, Grunert RR, Haff RF (1964). Antiviral activity of 1-adamantanamine (amantadine). Science.

[27] Peters NL, Oboler S, Hair C, Laxson L, Kost J, Meiklejohn G (1989). Treatment of an influenza A outbreak in a teaching nursing home. Effectiveness of a protocol for prevention and control. J Am Geriatr Soc.

[28] Schilling M, Gravenstein S, Drinka P (2004). Emergence and transmission of amantadine-resistant influenza A in a nursing home. J Am Geriatr Soc.

[29] Arden NH, Patriarca PA, Fasano MB (1988). The roles of vaccination and amantadine prophylaxis in controlling an outbreak of influenza A (H3N2) in a nursing home. Arch Intern Med.

[30] Gaillat J, Dennetiere G, Raffin-Bru E, Valette M, Blanc MC (2008). Summer influenza outbreak in a home for the elderly: application of preventive measures. J Hosp Infect.

[31] Bowles SK, Lee W, Simor AE (2002). Use of oseltamivir during influenza outbreaks in Ontario nursing homes, 1999–2000. J Am Geriatr Soc.

[32] Bush KA, McAnulty J, McPhie K (2004). Antiviral prophylaxis in the management of an influenza outbreak in an aged care facility. Commun Dis Intell.

[33] Babcock HM, Merz LR, Fraser VJ (2006). Is influenza an influenza-like illness? Clinical presentation of influenza in hospitalized patients. Infect Control Hosp Epidemiol.

[34] Babcock HM, Merz LR, Dubberke ER, Fraser VJ (2008). Case-control study of clinical features of influenza in hospitalized patients. Infect Control Hosp Epidemiol.

[35] Thursky K, Cordova SP, Smith D, Kelly H (2003). Working towards a simple case definition for influenza surveillance. J Clin Virol.

[36] Nichol KL (2006). Heterogeneity of influenza case definitions and implications for interpreting and comparing study results. Vaccine.

[37] Ferson MJ, Morgan K, Robertson PW, Hampson AW, Carter I, Rawlinson WD (2004). Concurrent summer influenza and pertussis outbreaks in a nursing home in Sydney, Australia. Infect Control Hosp Epidemiol.

[38] Mathur U, Bentley DW, Hall CB (1980). Concurrent respiratory syncytial virus and influenza A infections in the institutionalized elderly and chronically ill. Ann Intern Med.

[39] Goronzy JJ, Fulbright JW, Crowson CS, Poland GA, O'Fallon WM, Weyand CM (2001). Value of immunological markers in predicting responsiveness to influenza vaccination in elderly individuals. J Virol.

[40] Jefferson T, Del Mar CB, Dooley L (2011). Physical interventions to interrupt or reduce the spread of respiratory viruses. Cochrane Database Syst Rev.

[41] Win MK, Chow A, Chen M, Lau YF, Ooi EE, Leo YS (2010). Influenza B outbreak among influenza-vaccinated welfare home residents in Singapore. Ann Acad Med Singapore.

[42] Burette P, Bouuaert C, Melin P, Yane F, Brochier B, Giet D (2009). Influenza outbreak in a well-vaccinated nursing home population in Belgium. Acta Clin Belg.

[43] Dindinaud G, Potiron G, Agius G (1993). Influenza epidemic among a community of elderly people in spite of vaccination. Eur J Epidemiol.

[44] Drinka PJ, Gravenstein S, Krause P (1999). Non-influenza respiratory viruses may overlap and obscure influenza activity. J Am Geriatr Soc.

[45] Drinka PJ, Gravenstein S, Krause P, Nest L, Dissing M, Shult P (2000). Reintroduction of influenza A to a nursing building. Infect Control Hosp Epidemiol.

[46] Drinka PJ, Haupt T (2007). Emergence of rimantadine-resistant virus within 6 days of starting rimantadine prophylaxis with oseltamivir treatment of symptomatic cases. J Am Geriatr Soc.

[47] Goodman RA, Orenstein WA, Munro TF, Smith SC, Sikes RK (1982). Impact of influenza A in a nursing home. JAMA.

[48] Hall WN, Goodman RA, Noble GR, Kendal AP, Steece RS (1981). An outbreak of influenza B in an elderly population. J Infect Dis.

[49] Horman JT, Stetler HC, Israel E, Sorley D, Schipper MT, Joseph JM (1986). An outbreak of influenza A in a nursing home. Am J Public Health.

[50] Libow LS, Neufeld RR, Olson E, Breuer B, Starer P (1996). Sequential outbreak of influenza A and B in a nursing home: efficacy of vaccine and amantadine. J Am Geriatr Soc.

[51] Mast EE, Harmon MW, Gravenstein S (1991). Emergence and possible transmission of amantadine-resistant viruses during nursing home outbreaks of influenza A (H3N2). Am J Epidemiol.

[52] Surya P, Influenza A (1993). outbreaks–Louisiana, August 1993. MMWR Morb Mortal Wkly Rep.

[53] Morens DM, Rash VM (1995). Lessons from a nursing home outbreak of influenza A. Infect Control Hosp Epidemiol.

[54] Oguma T, Saito R, Masaki H (2011). Molecular characteristics of outbreaks of nosocomial infection with influenza A/H3N2 virus variants. Infect Control Hosp Epidemiol.

[55] Read CA, Mohsen A, Nguyen-Van-Tam JS, McKendrick M, Kudesia G (2000). Outbreaks of influenza A in nursing homes in Sheffield during the 1997–1998 season: implications for diagnosis and control. J Public Health Med.

[56] Degelau J, Somani SK, Cooper SL, Guay DR, Crossley KB (1992). Amantadine-resistant influenza A in a nursing facility. Arch Intern Med.

