# Supplementary material for: Influenza outbreak control practices and the effectiveness of interventions in long-term care facilities: a systematic review
Source: Influenza Other Respir Viruses. 2013 Nov 7;8(1):74–82. doi: 10.1111/irv.12203 (PMC3877675; doi:10.1111/irv.12203)
Supplement: Supplementary file 1 [file irv0008-0074-SD1.docx]

**Supplemental Table 1.** Influenza case and outbreak definitions.

| Article | Case definition | Outbreak definition |
| --- | --- | --- |
| Arden et al. (1988) | Temp>38.7°C & cough, coryza or sore throat | -- |
| Arroyo et al. (1984) | Acute respiratory tract infection during epidemic period | -- |
| Bowles et al. (2002) | ≥2 of the following: temp ≥37.5°C or <35.5°C, malaise/lethargy, cough, coryza/chest congestion, sore throat | -- |
| Burette et al. (2009) | -- | -- |
| Bush et al. (2004) | Onset of fever or cough or rhinitis & ≥1 other sx: sore throat, myalgia, headache, malaise, poor apetitie, chills | -- |
| Chang et al. (2008) | Temp>37.8°C, headache, myalgia, chills or fatigue & one of the following: cough, nasal congestion, sore throat | -- |
| Coles et al. (1989) | Temp>100.0°F (oral) & cough, sore throat, coryza or pneumonia | -- |
| Degelau et al. (1992) | Temp> 37.8°C & cough coryza or sore throat | -- |
| Dindinaud et al. (1993) | -- | -- |
| Drinka and Haupt (2007) | New respiratory symptom (cough, congestion, abnormal lung sounds, rhinorrhea, sore throat, resp rate >25 breaths per minute or deterioration in oximetry) & supporting symtpom (temp>99.5°F, headache, myalgia, malaise, naorexia, heart rate>100beats per minute, deterioration in daily activities, or mentation) | -- |
| Drinka et al. (1999) | Culture-confirmed influenza & acute respiratory symptoms including cough, sore throat or runny nose with or without fever | -- |
| Drinka et al. (2000) | Acute respiratory symptoms & culture-confirmed influenza A | -- |
| Ferson et al. (2004) | Acute onset of cough, fever & lethargy | -- |
| Gaillat et al. (2008) | Temp>38°C & cough and/or respiratory symptoms | -- |
| Goodman et al. (1982) | Temp>37.7°C (rectal) or cough | -- |
| Hall et al. (1981) | Temp>38°C (rectal) when no other causes identified | -- |
| Horman et al. (1986) | Temp≥37.8°C OR cough or chest congestion | -- |
| Infuso et al. (1996) | Temp≥38°C & cough and/or sputum production | -- |
| Lee et al. (2000) | ≥2 of the following: temp>37.4°C, cough, sore throat, myalgia, coryza, new wheezes or crackles | -- |
| Libow et al. (1996) | Temp>100.5°F & cough, coryza, lethargy, malaise and myalgia | -- |
| Mast et al. (1991) | Temp>38.0°C & cough, coryza or sore throat | -- |
| Mathur et al. (1980) | 4-fold rise in antibody titer by hemagglutination inhibition | ≥2 cases of upper respiratory illness within 72 hours on the same floor |
| Meiklejohn et al. (1989) | Age >65yrs AND temp>99.6°F & one of the following: respiratory symptoms of no explanation, pneumonia, or death from pneumonia w/in 1 month of onset | -- |
| MMWR (1993) | Temp≥100°F and cough | -- |
| Morens and Rash (1995) | Temp>37.8°C & cough, coryza or sore throat | -- |
| Murayama et al. (1999) | Culture-confirmed influenza virus, or serologic evidence of infection, or 4 of the following during outbreak or 6 of the following at any time: sudden onset, cough, rigors or chills, fever, prostration and weakness, myalgia, widespread aches and pain, no significant physical signs other than erythema of nasal mucous membrane and throat, and influenza in a close contact | overall attack rate of ≥10% within 7 day period |
| Oguma et al. (2011) | Temp≥37.8°C & cough, rhinorrhea, sneezing or sore throat | 2 or more influenza-like illness cases within 72 hours in any ward, in which ≥1 had a positive rapid detection test result |
| Parker et al. (2001) | -- | -- |
| Peters et al. (1989) | Temp>100°F & pharyngitis, myalgia and cough | -- |
| Read et al. (2000) | Acute onset of respiratory symptoms including cough, sore throat or myaglia with or without pyrexia >37.5°C | -- |
| Schilling et al. (1998) | Respiratory symptoms not accounted for by another disease process, no fever required | ≥10% residents in a unit with acute respiratory illness in 7-day window and influenza isolated in the facility during the previous 7 days |
| Schilling et al. (2004) | Culture-confirmed influenza A virus from incident respiratory illness | -- |
| Seale et al. (2009) | Fever & ≥2 of the following: cough, chills, prostration and weakness, myalgia | -- |
| Staynor et al. (1994) | ≥3 of the following: temp>38°C, cough, coryza, sore throat, lethargy or change in mental status, malaise | -- |
| Strassburg et al. (1986) | Temp≥100°F (rectal) & respiratory symptoms OR fever not ascribed to another cause | -- |
| Taylor et al. (1992) | Temp≥100°F & cough OR 4-fold increase in titer from complement fixation | -- |
| Win et al. (2010) | Fever & cough or running nose or sore throat & had contact with confirmed case during outbreak | -- |

*temp = temperature*

**Supplemental Table 2.** Number of influenza A outbreaks using one or two forms of chemoprophylaxis.

|  | Amantadine | Rimantadine | Oseltamivir | Zanamivir |
| --- | --- | --- | --- | --- |
| Amantadine | 9 | 4 | 5 | 1 |
| Rimantadine | -- | 2 | 0 | 8 |
| Oseltamivir | -- | -- | 9 | 0 |
| Zanamivir | -- | -- | -- | 2 |

*Outbreaks do not sum to 41 as an additional outbreak used amantadine, rimantadine and zanamivir.*

**Supplemental Table 3.** Staff and visitor policies for influenza outbreak control.

| Article | Staff restrictions | Visitor restrictions |
| --- | --- | --- |
| Schilling et al. (2004) | -- | Visitors asked to postpone visits if experiencing respiratory symptoms |
| Bush et al. (2004) | Do not report until >5 days post-symptom onset or after symptom resolution | Visitors restricted |
| Lee et al. (2000) | Restricted if ill | Visitors restricted |
| Morens and Rash (1995) | -- | Visitors screened for respiratory illness and not allowed onto ward if ill |
| Mast et al. (1991) | -- | Limitation of visitors |
| Ferson et al. (2004) | Cohorted with ill or well residents; ill staff report to supervisor with symptoms and take sick leave | Visitors reminded of hand hygiene and asked to not visit if experiencing respiratory symptoms |
| Read et al. (2000) | No temporary staff for duration of outbreak | Visitors advised of outbreak; elderly and parents of children asked to not visit |
| Arroyo et al. (1984) | -- | Sign posted at entrance to discourage visitors |
| Goodman et al. (1982) | -- | Limitation of visitors |
| Mathur et al. (1980) | -- | Visitors restricted |
| Staynor et al. (1994) | -- | Restriction of visitors |
| Seale et al. (2009) | Restricted to units where they were working | (Recommended by health department, action not reported) |
| Gaillat et al. (2008) | Advised to stay home until recovered | -- |
